# The Impact of Intensive Versus Standard Anthelminthic Treatment on Allergy-related Outcomes, Helminth Infection Intensity, and Helminth-related Morbidity in Lake Victoria Fishing Communities, Uganda: Results From the LaVIISWA Cluster-randomized Trial

**DOI:** 10.1093/cid/ciy761

**Published:** 2018-09-08

**Authors:** Richard E Sanya, Gyaviira Nkurunungi, Remy Hoek Spaans, Margaret Nampijja, Geraldine O’Hara, Robert Kizindo, Gloria Oduru, Prossy Kabuubi Nakawungu, Emmanuel Niwagaba, Elson Abayo, Joyce Kabagenyi, Christopher Zziwa, Josephine Tumusiime, Esther Nakazibwe, James Kaweesa, Fred Muwonge Kakooza, Mirriam Akello, Lawrence Lubyayi, Jaco Verweij, Stephen Nash, Ronald van Ree, Harriet Mpairwe, Edridah Tukahebwa, Emily L Webb, Alison M Elliott, LaVIISWA Trial Team, Richard Sanya, Margaret Nampijja, Harriet Mpairwe, Geraldine O’Hara, Barbara Nerima, Emily Webb, Remy Hoek Spaans, Lawrence Muhangi, Lawrence Lubyayi, Helen Akurut, Fatuma Nalukenge, Beatrice Mirembe, Justin Okello, Sebastian Owilla, Jonathan Levin, Stephen Nash, Milly Namutebi, Christopher Zziwa, Esther Nakazibwe, Josephine Tumusiime, Caroline Ninsiima, Susan Amongi, Grace Kamukama, Susan Iwala, Florence Akello, Mirriam Akello, Robert Kizindo, Moses Sewankambo, Denis Nsubuga, Stephen Cose, Linda Wammes, Prossy Kabuubi Nakawungu, Emmanuel Niwagaba, Gloria Oduru, Grace Kabami, Elson Abayo, Eric Ssebagala, Fred Muwonge Kakooza, Joyce Kabagenyi, Gyaviira Nkurunungi, Angela Nalwoga, Dennison Kizito, John Vianney Tushabe, Jacent Nassuuna, Jaco Verweij, Serge Versteeg, Ronald van Ree, Edward Tumwesige, Simon Mpooya, David Abiriga, Richard Walusimbi, Victoria Nannozi, Cynthia Kabonesa, James Kaweesa, Edridah Tukahebwa, Moses Kizza, Alison Elliott

**Affiliations:** 1 Immunomodulation and Vaccines Programme, Medical Research Council/Uganda Virus Research Institute and London School of Hygiene and Tropical Medicine Uganda Research Unit, Entebbe; 2 Department of Internal Medicine, College of Health Sciences, Makerere University, Kampala, Uganda; 3 Department of Clinical Research, London School of Hygiene and Tropical Medicine, United Kingdom; 4 Entebbe Hospital, Wakiso District Local Government, Uganda; 5 Vector Control Division, Ministry of Health, Kampala, Uganda; 6 Koome Health Centre III, Mukono District Local Government, Uganda; 7 Laboratory for Medical Microbiology and Immunology, St Elisabeth Hospital, Tilburg, The Netherlands; 8 Medical Research Council Tropical Epidemiology Group, Department of Infectious Disease Epidemiology, London School of Hygiene and Tropical Medicine, United Kingdom; 9 Academic Medical Centre, University of Amsterdam, Amsterdam, The Netherlands

**Keywords:** helminths, *Schistosoma mansoni*, mass drug administration, allergy-related disease, Africa

## Abstract

**Background:**

The prevalence of allergy-related diseases is increasing in low-income countries. Parasitic helminths, common in these settings, may be protective. We hypothesized that intensive, community-wide, anthelminthic mass drug administration (MDA) would increase allergy-related diseases, while reducing helminth-related morbidity.

**Methods:**

In an open, cluster-randomized trial (ISRCTN47196031), we randomized 26 high-schistosomiasis-transmission fishing villages in Lake Victoria, Uganda, in a 1:1 ratio to receive community-wide intensive (quarterly single-dose praziquantel plus albendazole daily for 3 days) or standard (annual praziquantel plus 6 monthly single-dose albendazole) MDA. Primary outcomes were recent wheezing, skin prick test positivity (SPT), and allergen-specific immunoglobulin E (asIgE) after 3 years of intervention. Secondary outcomes included helminths, haemoglobin, and hepatosplenomegaly.

**Results:**

The outcome survey comprised 3350 individuals. Intensive MDA had no effect on wheezing (risk ratio [RR] 1.11, 95% confidence interval [CI] 0.64–1.93), SPT (RR 1.10, 95% CI 0.85–1.42), or asIgE (RR 0.96, 95% CI 0.82–1.12). Intensive MDA reduced *Schistosoma mansoni* infection intensity: the prevalence from Kato Katz examinations of single stool samples from each patient was 23% versus 39% (RR 0.70, 95% CI 0.55–0.88), but the urine circulating cathodic antigen test remained positive in 85% participants in both trial arms. Hookworm prevalence was 8% versus 11% (RR 0.55, 95% CI 0.31–1.00). There were no differences in anemia or hepatospenomegaly between trial arms.

**Conclusions:**

Despite reductions in *S. mansoni* intensity and hookworm prevalence, intensive MDA had no effect on atopy, allergy-related diseases, or helminth-related pathology. This could be due to sustained low-intensity infections; thus, a causal link between helminths and allergy outcomes cannot be discounted. Intensive community-based MDA has a limited impact in high-schistosomiasis-transmission fishing communities, in the absence of other interventions.

**Clinical Trials Registration:**

ISRCTN47196031.

## Teaser

In a cluster-randomized trial of intensive versus standard anthelminthic treatment, intensive treatment reduced *Schistosoma mansoni* intensity and hookworm prevalence, but did not effect atopy, allergy-related diseases, or helminth-related pathology. Additional interventions are required to reduce transmission in schistosomiasis hot spots.

The prevalence of allergy-related diseases (ARD), such as eczema, rhinitis, and asthma, increased rapidly in high-income countries in the twentieth century [1] and is now increasing in tropical, low-income countries (LICs) [2]. Nevertheless, populations in LICs, particularly in rural settings, remain relatively protected [3]. Understanding this phenomenon is crucial to elucidating the causes and improving prevention of ARD.

By contrast, LICs carry the largest burden of parasitic helminth infections: these are associated with some severe and much subtle morbidity [4, 5]. Major anthelminthic mass drug administration (MDA) has taken place in the last decade but, although prevention of severe helminth-induced morbidity is important, wider benefits [6] and the sustainability of helminth control by MDA [7, 8] have been questioned.

Certain helminth antigens are highly homologous to allergens; immunoglobulin (Ig)-E and the atopic pathway are presumed to have evolved to protect mammals against such organisms [9]. Parasitic helminths must modulate such responses to survive within mammalian hosts. Animal and human epidemiological and in vitro studies indicate that, through bystander effects of such immunomodulation, chronic helminth infection protects against atopy and ARD [10]. If helminths protect against ARD, MDA programs may adversely affect these outcomes. Observational studies, many of which indicate an inverse association between helminths and ARD, are subject to confounding and reverse causation; therefore, several groups have investigated the effects of anthelminthic treatment on ARD in clinical trials. Some studies show increased atopy after anthelminthic intervention, but 2 large, school-based, individually-randomized intervention trials focusing on soil-transmitted helminths (STH) reported no effect on atopy or ARD [11, 12]. A recent household-randomized trial of intensive albendazole for STH showed no effect on ARD, but upregulated pro-inflammatory responses and reduced immunoregulatory molecules [13].

East African fishing communities bear an intense schistosomiasis burden [14]. During a *Schistosoma mansoni* infection, adult worms reside in mesenteric blood vessels and eggs are excreted through intestinal mucosa, causing intestinal and tissue (notably liver) pathology [5]. *Schistosoma* infection has shown even stronger inverse associations with atopy than STH [15] and there is evidence of increased SPT reactivity with treatment [16], but no large-scale randomized trial on the allergy-related effects of intensively treating schistosomiasis has been conducted.

We undertook the Lake Victoria Island Intervention Study on Worms and Allergy-related diseases (LaVIISWA; ISRCTN47196031) [17], a cluster-randomized trial of extended (3-year) intensive versus standard anthelminthic intervention, to assess the causal role of helminths in allergy-related outcomes and the benefits of intensive intervention for helminth-related morbidity in a schistosomiasis hot spot.

## METHODS

### Design and Setting

This was a 2-arm, open, cluster-randomized trial of intensive versus standard anthelminthic treatment conducted among fishing villages in the Koome islands, Lake Victoria, Uganda, between September 2012 and August 2016. The protocol has been published previously [17]. We randomized 26 villages 1:1 to either intensive or standard intervention. Village-level cluster-randomization aimed to minimize contamination from reinfection by untreated neighbours. Before the study, annual praziquantel treatment was offered to these communities, but hampered by logistics. In our baseline survey, 17% participants reported treatment in the prior year [17].

### Interventions

Standard intervention, based on the Uganda Ministry of Health guidelines, was annual single dose praziquantel at 40 mg/kg (Cipla; CSPC OUYI Pharmaceuticals, India; AGOG Pharma, India) to community members ≥94 cm, as estimated by a height pole, plus 6 monthly single dose albendazole at 400 mg (CSPC OUYI Pharmaceuticals, India; AGOG Pharma, India; Medreich, India) to all aged ≥1 year. Intensive intervention was quarterly single dose praziquantel at 40 mg/kg to individuals ≥60 cm (to allow treatment of younger children) [18], as estimated by an extended height pole, plus quarterly triple dose albendazole (400 mg daily for 3 days) to all aged ≥1 year. Pregnant women were included in both arms, receiving single dose albendazole [19, 20].

Treatment, distributed house-to-house in collaboration with the Uganda Ministry of Health Vector Control Division, was directly observed and documented against household registers, with the exception of post–day 1 albendazole in the intensive arm.

### Participants and Surveys

Leaders of all 27 Koome fishing villages gave written consent for their village’s participation. Allocated interventions were given to all community members (of eligible age and height) unless they were absent, sick, or refused.

Household-based surveys were conducted at baseline [21] and after 3 years of intervention. All primary and most secondary outcomes were assessed in both baseline and outcome surveys. Smaller surveys were conducted at years 1 and 2 to assess helminth trends (Supplementary Figure). Separate random household samples were selected for each survey (overlap was possible). There was no individual participant follow-up. Surveys were conducted immediately prior to respective quarterly treatments.

Household registers were updated before each survey. Villages generally comprised an intensely-populated center and a scattered periphery. Peripheral households were excluded from surveys to avoid contamination from neighboring villages, but received allocated interventions.

Baseline survey methods (previously reported) were similar to the 3-year outcome survey described below [21]. For interim surveys, stool and blood samples were collected from community members selected using a 2-stage method: 1 person was randomly selected from each of 15 randomly-selected households per village. For the 3-year survey, 70 households per village were randomly selected using a Stata program (StataCorp, College Station). In the selected households, all members ≥1 year were invited to participate. Household heads gave permission for household participation and provided the demographic details (age, sex) of all household members. Written informed consent was obtained from all adults and emancipated minors and from parents/guardians for children, with additional assent from children ≥8 years. For each participant, a questionnaire was completed; an examination and SPT were performed; and blood, urine, and 1 stool sample were obtained. Abdominal ultrasonography was performed on children.

### Outcomes

Primary outcomes were recent (last 12 months) self-reported wheezing, stratified by age (<5 years, ≥5 years); SPT positivity to mites (*Dermatophagoides* mix, *Blomia tropicalis*) and German cockroaches (*Blattella germanica*); and allergen-specific IgE (asIgE) to *Dermatophagoides* and German cockroaches (common allergens in Uganda [22]). Secondary outcomes were visible flexural dermatitis (assessed using standardized procedures); helminth infections; haemoglobin; growth (height-for-age, <20 years; weight-for-age, <11 years; and weight-for-height, <6 years, z-scores); and hepatosplenomegaly (by palpation). An additional secondary outcome, schistosomiasis-related liver and spleen morbidity assessed by abdominal ultrasonography (<18 years), was included after trial interventions commenced, when additional funding became available. Exploratory outcomes were recent urticaria and rhinitis. For logistical reasons, we could not provide infant vaccines ourselves or obtain post-immunization samples at consistent timepoints, so planned vaccine response secondary outcomes are not reported. Details on outcome ascertainment are provided (Supplementary Methods).

### Randomization

At a public ceremony, 1 village was randomly selected for piloting while 26 were randomized 1:1, using restricted randomization to balance village size, prior praziquantel treatment, and distance from the sub-county health center [17] (Supplementary Methods).

### Statistical Methods

For the outcome survey, we planned to sample 1540 individuals per arm (Supplementary Methods). Data were analyzed using Stata v14.0. Baseline characteristics were tabulated. Characteristics of survey participants were compared with those of non-participants by chi-squared tests. Treatment uptake was calculated both by village and treatment round as the number of people receiving treatment divided by the total number of residents.

Trial analyses were done at the cluster level. Crude and adjusted analyses (adjusting for sex, age, and the corresponding baseline summary measure of the outcome, where available) were performed. For binary outcomes, risk ratios (RRs) were calculated as the mean of the intensive-arm cluster proportions divided by the mean in the standard arm, with 95% confidence intervals (CI) calculated using a Taylor series approximation for the standard error and *P* values from unpaired *t*-tests. Where the distribution of cluster proportions was skewed, the log-cluster proportions were compared and the results were back-transformed. A 2-stage approach was used for adjusted analyses [23] (Supplementary Methods).

For continuous outcomes, intervention effects were quantified as the differences in mean outcome between trial arms, with 95% CIs calculated using the *t*-distribution. Non–normally distributed continuous outcomes were log-transformed and the results were back-transformed to obtain geometric mean ratios. For ordered categorical outcomes, a proportional-odds model was used.

Trial analyses were conducted in 2 populations: the primary analysis population (intention-to-treat population) included all individuals. The secondary analysis population comprised all individuals who had lived in their village throughout (or were born into their village during) the intervention period (per-protocol analysis).

Using a cluster-level approach [24], we conducted post hoc subgroup analyses by age group (<4 years, ≥4 years) for the primary outcomes to assess whether intervention effects differed among those exposed to differential anthelminthic interventions from birth.

### Ethics Statement

Ethical approval was given by the Uganda Virus Research Institute (GC127), Uganda National Council for Science and Technology (HS 1183), and London School of Hygiene and Tropical Medicine (6187).

## RESULTS

### Participants and Intervention Uptake

Characteristics assessed in the baseline survey (October 2012–July 2013) were balanced between trial arms, with the exception that, compared to villages in the intensive arm (intensive villages), villages in the standard arm (standard villages) had fewer public toilets but contained more households with private toilets [17].

**Figure 1.**
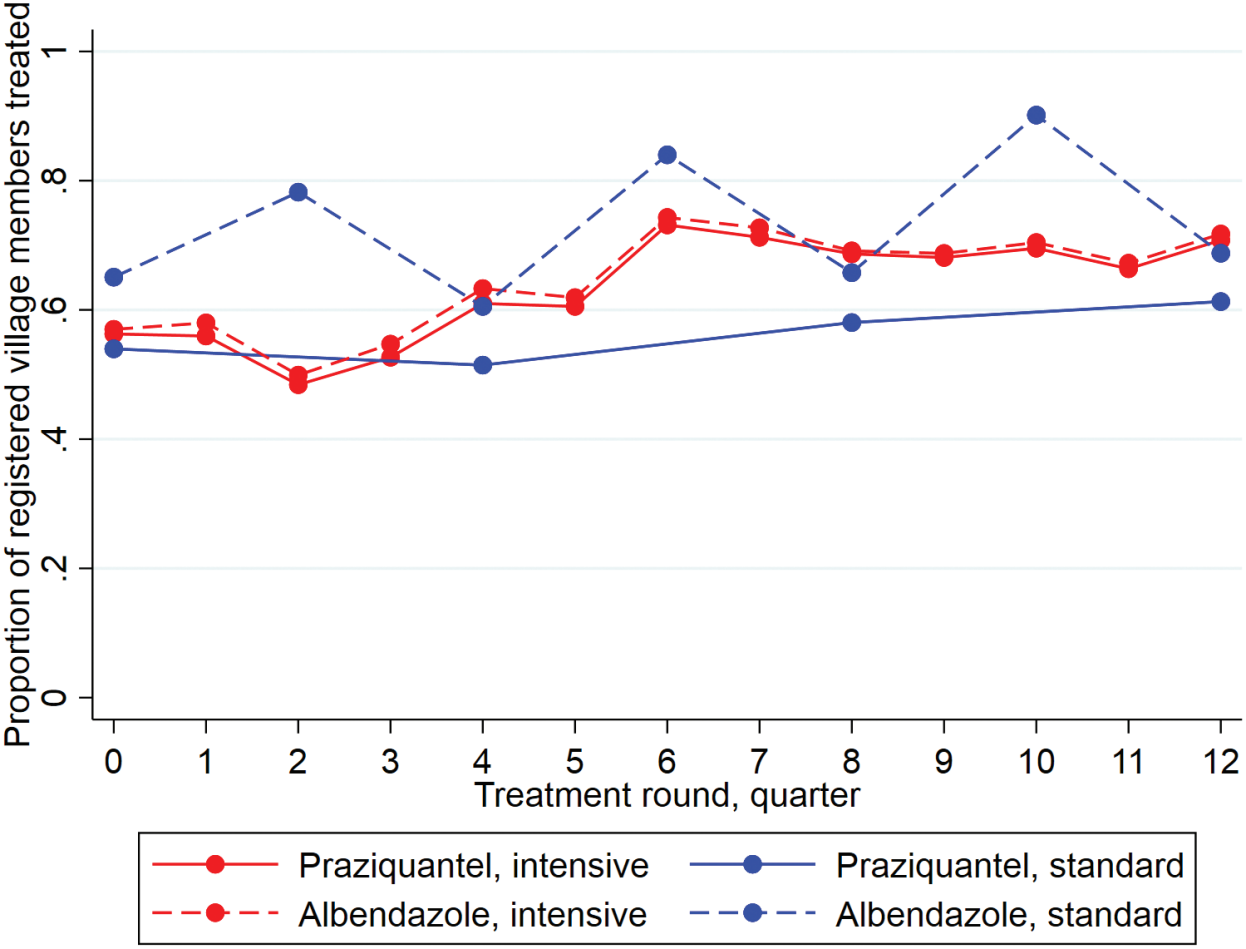
Praziquantel and albendazole treatment coverage, by trial arm and treatment round.

Figure 1 summarizes the treatment uptake. Both the praziquantel and albendazole uptake increased during the trial. The mean uptake per round was 63% for praziquantel and 64% for albendazole (intensive villages), compared to 56% and 73% (standard villages). In standard villages, the albendazole uptake was lower in the treatment rounds where praziquantel treatment was also given. Reported receipt of ≥1 dose of praziquantel in the preceding year was higher in intensive, compared to standard, villages (93% versus 75%, respectively). Reported receipt of ≥1 dose of albendazole was universally high (99% versus 98%, respectively).

**Figure 2.**
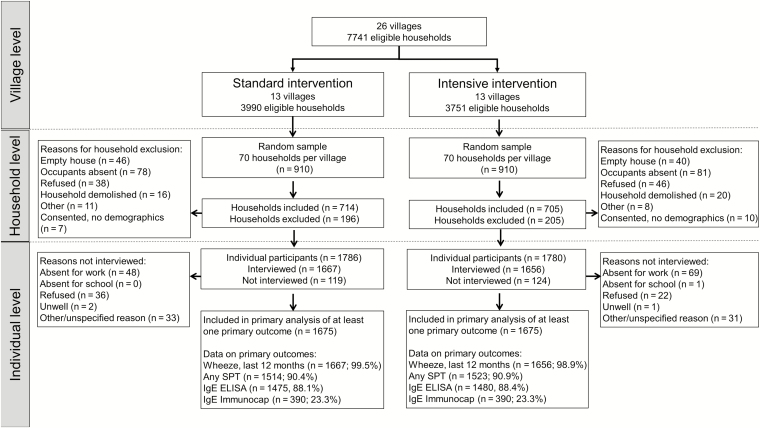
Trial flowchart. Abbreviations: ELISA, enzyme-linked immunosorbent assay; IgE, immunoglobulin E; SPT, skin prick test positivity.

Between September 2015 and August 2016, 70 households from each village were randomly selected for the outcome survey (Figure 2): 84 (5%) households refused to participate, 17 (1%) consented but no demographic data were captured, and 300 (17%) had no members that could be contacted. The remaining 1419 participating households contained 3566 residents aged ≥1 year. Overall, 3350 (94%) household members provided data for at least 1 primary outcome (recent wheezing 3323 [99%], SPT 3037 [91%], IgE 2955 [88%]), with numbers balanced between trial arms (Figure 2). Further details of participant characteristics are provided (Supplementary Material).

Outcome survey participant characteristics were comparable between trial arms (Table 1). Only 8 villages had access to any non-lake water supply, with public toilets available in 11 villages and private toilet access limited. The participant median age was 24 years (interquartile range 8–34) and 52% were male. Most participants (71%) had lived in their village throughout the trial. Migration between trial arms was low (1.5%). The adult human immunodeficiency virus prevalence was 22% and the reported maternal history of allergy, eczema, or asthma was 16%.

**Table 1.** Characteristics of Outcome Survey Participants

Cluster-level Characteristics	Standard Arm		Intensive Arm	
	(n = 13)		(n = 13)	
Mean no. of households per village (range)	307	(124–882)	289	(87–544)
Mean no. of participating households (range)	55	(48–63)	54	(48–64)
Mean no. of individuals resident in participating households (range)	137	(89–161)	137	(85–177)
Mean no. of individuals included in analysis (range)	129	(84–150)	129	(79–169)
Villages with any public toilets	5	38%	6	46%
Median no. of public toilets (range)	0	(0–16)	0	(0–20)
Median no. of private toilets (range)	8	(0–59)	3	(1–29)
Water supply other than lake	3	23%	5	38%
Piped water	2	67%	2	40%
River or open spring	1	33%	2	40%
Open well	0	0%	1	20%
**Household-level characteristics**	**(n = 714)**		**(n = 705)**	
Median no. of household members (IQR)	2	(1–3)	2	(1–3)
**Individual-level characteristics**	**(n = 1675)**		**(n = 1675)**	
Sex, male	881	53%	857	51%
Age in years, grouped				
0–4	283	17%	264	16%
5–9	173	10%	219	13%
10–14	66	4%	115	7%
15–19	102	6%	79	5%
20–24	212	13%	179	11%
25–29	239	14%	216	13%
30–34	198	12%	211	13%
35–39	175	10%	140	8%
40–44	86	5%	106	6%
45+	141	8%	146	9%
Place of birth (mv 9, 19)				
This fishing village	439	26%	477	29%
Other fishing village	48	3%	20	1%
Other rural village	1021	61%	1002	61%
Town	127	8%	127	8%
City	31	2%	30	2%
Has remained in village during intervention period (mv 9, 19)	1190	71%	1170	71%
Has lived in other trial arm during intervention period (mv 9, 19)	18	1%	32	2%
Maternal history of allergic diseases (mv 9, 19)				
No history	1193	72%	1204	73%
History of asthma, eczema or allergies	258	15%	266	16%
Don’t know	215	13%	186	11%
Paternal history of allergic diseases (mv 9, 19)				
No history	1248	75%	1244	75%
History of asthma, eczema or allergies	145	9%	155	9%
Don’t know	273	16%	257	16%
Occupation, grouped by type (mv 8, 19)				
Child, not at school	289	17%	275	17%
Student	257	15%	345	21%
Housewife	120	7%	101	6%
Fishing or lake related	564	34%	467	28%
Shops, saloons, artisans, service providers	118	7%	102	6%
Bars, restaurants, food providers, entertainment	114	7%	103	6%
Agricultural, lumbering, charcoal	157	9%	201	12%
Professional	11	1%	19	1%
Unemployed	37	2%	43	3%
Treated with albendazole in the last 12 months (mv 360, 253)	1291	98%	1404	99%
Treated with praziquantel in the last 12 months (mv 355, 253)	989	75%	1318	93%
Malaria treatment with coartem (mv 190, 167)	708	42%	747	45%
Malaria positivity by blood smear (*Plasmodium falciparum*; mv 213, 214)	50	3%	52	4%
**Individuals aged 13 years and over**	**(n = 1176)**		**(n = 1112)**	
Frequency of lake contact (mv 9, 19)				
Every day	911	78%	776	71%
Almost every day	126	11%	147	13%
Once a week	95	8%	124	11%
Once a month	30	3%	35	3%
Less than once a month	4	0%	10	1%
Never	1	0%	1	0%
**Individuals aged 18 years and over**	**(n = 1116)**		**(n = 1041)**	
HIV+ (mv 173, 176)	192	20%	198	23%
HIV+ on ART	90	47%	103	52%
HIV+ not on ART	93	48%	90	45%
HIV+ not known if receiving ART	9	5%	5	3%

Numbers for mvs are in the standard and intensive arms, respectively.

Abbreviations: ART, antiretroviral therapy; HIV, human immunodeficiency virus; IQR, interquartile range; mv, missing values; no., number.

### Impact of Intensive Versus Standard Anthelminthic Treatment on Primary Outcomes

The prevalence of reported wheezing among ≥5-year-olds was 3%, with little difference between trial arms (Table 2). There were 9 individuals <5 years old who reported wheezing; no formal analysis was done for this outcome. Regarding atopy, 19% participants had a positive SPT to ≥1 allergen. Of those tested using ImmunoCAP, 54% were positive (IgE > 0.35kUa/L) for either cockroach or dust mite allergens. Enzyme-linked immunosorbent assay (ELISA) and ImmunoCAP results were positively correlated for both dust mites and cockroaches (Spearman’s correlation coefficient 0.32 and 0.29, respectively). There was no effect of intensive versus standard treatment on atopy (by SPT or IgE; Table 2). For all primary outcomes, there remained little evidence of a difference between trial arms in the per-protocol analysis (Supplementary Table 1) or among age groups (Supplementary Table 2), although RRs for SPT responses to individual allergens increased in both the per-protocol analysis and in children <4 years old.

**Table 2.** Impact of Intensive Versus Standard Anthelminthic Treatment on Primary Outcomes

Outcome	n/N (%)/Geometric Mean		Unadjusted		Adjusted for Outcome at Baseline by Age and Sex^a^	
	Standard	Intensive	RR/GMR (95% CI)	*P* Value	RR/GMR (95% CI)	*P* Value
Wheeze (age ≥5 years)^b^	44/1384 (3.2%)	43/1392 (3.1%)	1.06 (0.61–1.87)	.82	1.11 (0.64–1.93)	.69
Wheeze (age <5 years)	6/284 (2.1%)	3/264 (1.1%)				
Atopy (SPT)						
SPT positivity to any allergen	273/1514 (18.0%)	303/1523 (19.9%)	1.09 (0.83–1.44)	.51	1.10 (0.85–1.42)	.46
SPT positivity to *Dermatophagoides*	162/1514 (10.7%)	164/1523 (10.8%)	0.98 (0.72–1.35)	.92	1.00 (0.74–1.36)	.99
SPT positivity to *Blomia tropicalis*	102/1514 (6.7%)	127/1522 (8.3%)	1.26 (0.83–1.90)	.26	1.27 (0.85–1.91)	.22
SPT positivity to German cockroach	156/1513 (10.3%)	194/1522 (12.8%)	1.24 (0.87–1.77)	.20	1.22 (0.87–1.71)	.21
Atopy (IgE detected by ImmunoCAP)						
*Dermatophagoides* or cockroach positivity (>0.35 kUa/L)	214/390 (54.9%)	210/390 (53.9%)	0.97 (0.83–1.13)	.67	0.96 (0.82–1.12)	.60
*Dermatophagoides* positivity (asIgE > 0.35 kUa/L)	134/390 (34.4%)	130/390 (33.3%)	0.95 (0.76–1.20)	.67	0.96 (0.77–1.19)	.68
German cockroach positivity (asIgE > 0.35 kUa/L)	201/390 (51.5%)	192/390 (49.2%)	0.94 (0.80–1.11)	.47	0.94 (0.79–1.11)	.42
Concentration of asIgE to *Dermatophagoides* (kUa/L)^c^	GM: 0.158	GM: 0.129	0.78 (0.51–1.17)	.22	0.77 (0.52–1.13)	.17
Concentration of asIgE to German cockroach (kUa/L)^c^	GM: 0.342	GM: 0.289	0.82 (0.55–1.22)	.31	0.81 (0.55–1.20)	.28
Atopy (IgE detected by in house ELISA)						
Concentration of asIgE to *Dermatophagoides*^d^	GM: 60.3	GM: 73.8	1.13 (0.36–3.50)	.83	1.17 (0.39–3.51)	.78
Concentration of asIgE to German cockroach^d^	GM: 72.4	GM: 161.0	1.98 (0.59–6.63)	.25	1.51 (0.45–5.04)	.49

Abbreviations: asIgE, allergen-specific IgE; CI, confidence interval; ELISA, enzyme-linked immunosorbent assay; GM, geometric mean; GMR, geometric mean ratio; IgE, immunoglobulin E; RR, risk ratio; SPT, skin prick test positivity.

^a^Atopy outcomes assessed by IgE were adjusted for age and sex only.

^b^For this outcome, a natural log transformation was applied to village-level proportions to correct skewed distributions, and the data in parentheses are the geometric means of village proportions.

^c^Log10(+0.001) transformation at the individual level.

^d^Log10(+1) transformation at the individual level.

### Impact of Intensive Versus Standard Anthelminthic Treatment on Secondary and Exploratory Outcomes

**Figure 3.**
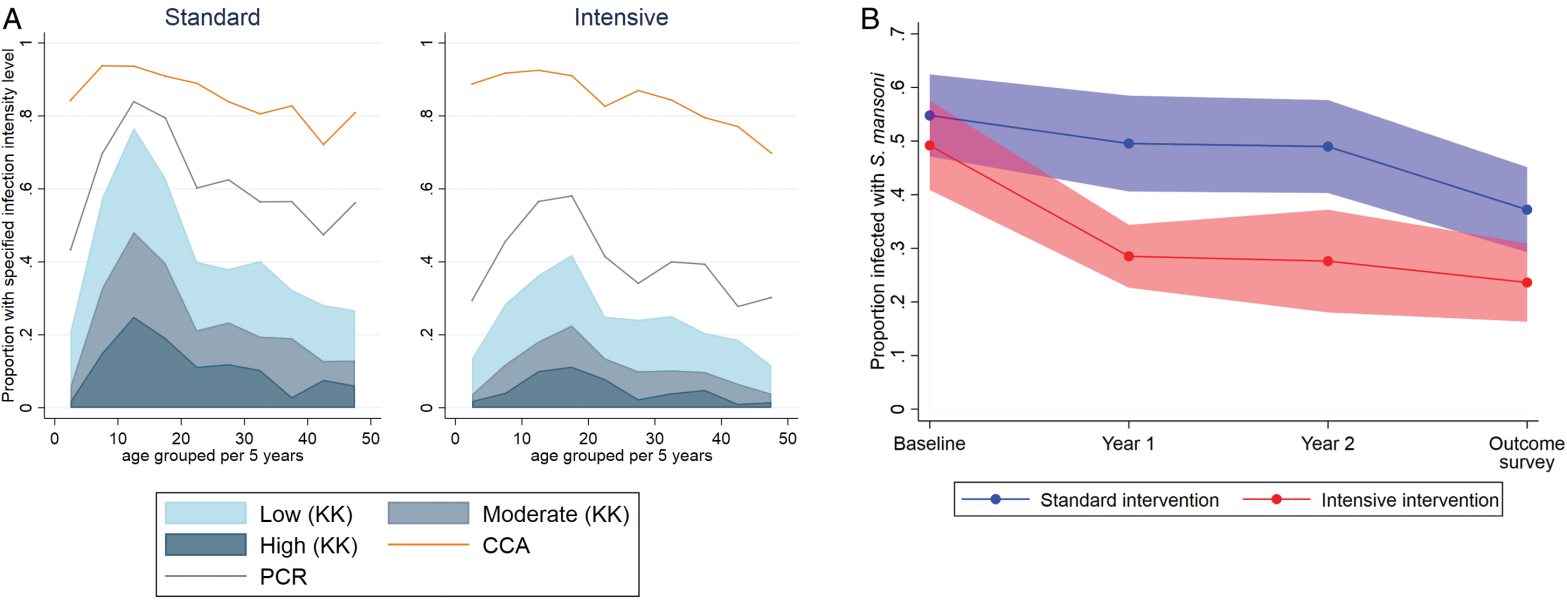
(*A*) Intensity of schistosomiasis infection in the outcome survey, by age group and trial arm, with prevalence assessed by KK examination of a single stool sample, PCR, and urine CCA. (*B*) Prevalence of *Schistosoma mansoni* infection over time (pre-intervention baseline survey, interim survey at 1 year, interim survey at 2 years, outcome survey at 3 years), by trial arm. Data are shown as the mean of village prevalences over time +/- 95% confidence intervals, assessed using KK analysis of a single stool sample (with duplicate slides) at each time point. Abbreviations: CCA, circulating cathodic antigen; KK, Kato Katz; PCR, polymerase chain reaction.

The *Schistosoma mansoni* infection prevalence was lower in the intensive-treatment villages when assessed by stool Kato Katz (23% versus 39%, respectively; adjusted RR 0.70, 95% CI 0.55–0.88; Table 3) and stool polymerase chain reactions (39% versus 60%, respectively; adjusted RR 0.76, 95% CI 0.65–0.88), but urine circulating cathodic antigen positivity remained high and similar across trial arms (both 85%; Table 3), indicating that the intensive treatment was more effective than the standard treatment in reducing heavy-intensity *Schistosoma* infections, particularly in younger age groups, but had little impact on the light-infection prevalence (Figure 3). The incidence of *Schistosoma* infections was lower in both trial arms, compared to baseline, with 49% and 23% pre- and post-intervention in the intensive arm, respectively, and 56% and 39% in the standard arm, respectively. Our interim survey data suggested a greater initial reduction in intensive villages, which then plateaued, and a gradual reduction in standard villages (Figure 3). The STH prevalence was relatively low. The intensive treatment reduced hookworm prevalence; no significant reductions were seen for other nematodes (among all participants; Table 3). There was no impact of the intensive versus standard treatment on anthropometric or clinical outcomes, including hepatosplenomegaly, assessed either by palpation (Table 3) or ultrasound (among children; Supplementary Table 3). The per-protocol analysis did not yield any hitherto-unseen differences (Supplementary Table 4).

**Table 3.** Impact of Intensive Versus Standard Anthelminthic Treatment on Helminths, Clinical Outcomes, Hepatosplenomegaly by Palpation, and Anthropometry

Outcome	n/N (%)/Arithmetic Mean		Unadjusted		Adjusted for Outcome at Baseline by Age and Sex	
	Standard	Intensive	RR/Mean Difference (95% CI)	*P* Value	RR/Mean Difference (95% CI)	*P* Value
Helminth infections						
*Schistosoma mansoni*, stool Kato Katz	523/1355 (38.6%)	323/1396 (23.1%)	0.64 (0.43–0.94)	.02	0.70 (0.55–0.88)	.003
*Schistosoma mansoni,* stool PCR	797/1353 (59.9%)	541/1394 (38.8%)	0.68 (0.52–0.89)	.007	0.76 (0.65–0.88)	.001
*Schistosoma mansoni*, urine CCA	1229/1444 (85.1%)	1216/1435 (84.7%)	0.99 (0.91–1.08)	.85	1.00 (0.93–1.08)	.93
Hookworm, stool PCR^a^	147/1353 (10.9%)	112/1394 (8.0%)	0.54 (0.28–1.02)	.06	0.55 (0.31–1.00)	.05
*Strongyloides stercoralis*, stool PCR	112/1353 (8.3%)	78/1394 (5.6%)	0.74 (0.50–1.11)	.14	0.78 (0.54–1.14)	.21
*Trichuris trichiura*, stool Kato Katza	137/1355 (10.1%)	108/1396 (7.7%)	0.91 (0.40–2.09)	.82	0.85 (0.48–1.50)	.55
*Ascaris lumbricoides*, stool Kato Katz	11/1355 (0.8%)	3/1396 (0.2%)				
Clinical outcomes						
Visible flexural dermatitis	1/1558 (0.1%)	4/1553 (0.3%)				
Haemoglobin	14.0	13.9	-0.06 (-0.37–0.25)	.70	0.00 (-0.24–0.25)	.97
Anthropometry						
Height-for-age z-score, age 1–19 years	-0.48	-0.49	-0.01 (-0.20–0.19)	.95	0.02 (-0.16–0.20)	.83
Weight-for-age z-score, age 1–10 years	-0.06	-0.17	-0.11 (-0.31–0.09)	.27	-0.05 (-0.23–0.12)	.52
Weight-for-height z-score, age 1–5 years	0.15	0.19	-0.09 (-0.43–0.26)	.62	-0.06 (-0.40–0.28)	.72
Hepatosplenomegaly, palpation						
Hepatomegaly, palpation	100/1546 (6.5%)	98/1546 (6.3%)	0.97 (0.71–1.32)	.83	0.96 (0.70–1.32)	.80
Splenomegaly, palpation	87/1549 (5.6%)	63/1547 (4.1%)	0.73 (0.43–1.25)	.20	0.70 (0.43–1.15)	.13
Hepatosplenomegaly, palpation^a^	22/1548 (1.4%)	14/1548 (0.9%)	0.85 (0.52–1.39)	.49	0.78 (0.47–1.30)	.33
Reported clinical outcomes (exploratory)						
Urticaria, last 12 months	162/1667 (9.7%)	172/1656 (10.4%)	1.06 (0.86–1.30)	.59	1.06 (0.88–1.27)	.51
Rhinitis, last 12 months	78/1667 (4.7%)	74/1656 (4.5%)	1.02 (0.73–1.42)	.92	1.00 (0.74–1.36)	.99

Abbreviations: CCA, circulating cathodic antigen; CI, confidence interval; PCR, polymerase chain reaction; RR, risk ratio.

^a^For this outcome, a natural log transformation was applied to village-level proportions to correct skewed distributions.

### Serious Adverse Events

In total, 77739 praziquantel treatments and 102219 albendazole treatments were given. There were 4 serious adverse events reported (2 in each trial arm), all among adults and within 2 days of treatment: gastrointestinal symptoms leading to hospitalization (1) or requiring intravenous fluids (1); abdominal pain and vaginal bleeding in a non-pregnant woman (1); and vaginal bleeding 1 day after treatment in a pregnant woman, followed by delivery 3 days later (probably premature) and subsequent neonatal death (1). Clinic records suggested that this last woman had concurrent malaria, but this remained unconfirmed.

## DISCUSSION

We report the first trial to address the community-level effects of intensive anthelminthic MDA in a high–*Schistosoma mansoni*–transmission setting. After 3 years, we found no effect of an intensive, compared to standard, intervention on allergy-related or helminth-associated disease outcomes. Intensive, compared to standard, treatment with praziquantel achieved a substantial reduction in *S. mansoni* intensity, most marked after 1 year, but infection remained almost universal. Intensive, compared to standard, treatment with albendazole achieved a modest reduction in hookworm prevalence, but had little impact on *Trichuris* or *Strongyloides*.

The prevalence of wheezing was lower than anticipated based on previous reports [25], limiting power for this outcome. However, the understanding of “wheeze” in our study communities was poor: there are no words for wheeze or asthma in the vernacular and asthma is rare. That said, there was no effect of the intensive intervention on wheezing, and no increase in wheezing during the intervention (5% at baseline [13], 3% after 3 years). These results provide reassurance that anthelminthic MDA is unlikely to have an immediate adverse effect on asthma among high-schistosomiasis-transmission communities, although no conclusions can be drawn on the impact of effective, universal *S. mansoni* removal.

SPT positivity was common. There was no increase in SPT positivity during the intervention (19% at baseline [13], 18% and 20% in the standard and intensive arms, respectively, after 3 years). There was a suggestion, especially in the per-protocol analysis and in under–4 year olds, that SPT responses increased with intensive treatment. This could be a chance finding, since a substantial number of (planned) statistical tests were conducted. This warrants more detailed investigation, as it may presage the emergence of increased atopy and ARD when helminth infections are more completely cleared. The effect of treatment may have differed based on pre-treatment infection intensity [26]. We could not assess this hypothesis, because our study was not a cohort of individual subjects.

Despite our emphasis on schistosomiasis and on long-term, community-based intervention, our results accord with previous, shorter-term trials focussing on STH [10]. However, it seems premature to conclude that high helminth prevalence has no causal link with the low ARD prevalence in LICs, given the strong effects and demonstrated mechanisms in animal models and experiments using human samples in vitro [27].

The most obvious explanation for a lack of impact on allergy-related (or helminth-associated) diseases is a failure to clear helminth infections. All villages were continuously exposed to *S. mansoni*–infested lake water because of a lack of alternative safe water, an involvement in fishing, and open defecation due to a scarcity of latrines. Although single- and first-dose treatments were directly observed, compliance was imperfect; albendazole uptake in the standard arm was lower in the rounds where praziquantel was given, indicating that villagers were averse to the praziquantel side effects. Furthermore, we cannot rule out the possible role of reduced drug efficacy [28, 29]. However, as a differential effect on helminth intensity was achieved, particularly for schistosomiasis, our results cast doubt on the extent to which intensity reduction (without elimination) substantially modifies the overall immunological or pathological effects in high-schistosomiasis-transmission settings.

Other factors contributing to the lack of impact on allergy-related outcomes may include the long-term immunological effects of helminth exposure through a persistence of antigen or through epigenetic changes in immunological pathways [30]. Also, in tropical, low-income settings, numerous other exposures—including immunomodulating infections such as malaria, exposure to dirt and domestic animals, or the microbiome profile—may impact allergy-related outcomes, such that modifying helminth exposure alone may have a limited impact [31].

A recent meta-analysis examined the effects of schistosomiasis treatments on related morbidity [32]. The results indicated wide-ranging benefits, with an increased impact when egg reduction rates were greatest and, for anemia and chronic morbidities, when treatments were repeated over periods of greater than 24 months. Thus, we were disappointed that, despite a differential reduction in schistosome intensity, we found no evidence that 3 years of intensive (compared to standard) intervention achieved improvement in any morbidity measure. This adds to the evidence base showing the limited effects of MDA on such outcomes at the community-level. We identified surprisingly little severe *Schistosoma*-related morbidity in this community, despite intense infections, consistent with earlier work from Lake Victoria communities; it is possible that intensive interventions would have a greater benefit in settings (such as Lake Albert) where severe pathology is more common [33].

Our experience emphasizes that MDA may struggle to eliminate helminths as a public health problem, especially in high-transmission environments. The substantial decline in *S. mansoni* infections (as measured by Kato Katz) achieved in year 1 led us to hope that intensive intervention could make an important contribution to schistosomiasis control in these challenging hot spots. The subsequent plateau and persistent infection (as measured by by circulating cathodic antigen) were disheartening. This phenomenon (a large drop in prevalence, followed by a subsequent plateau) has also been reported in Kenyan districts bordering Lake Victoria [34]. Besides reinfection, the possibility of selection for praziquantel-resistant or -tolerant strains is of concern [35]. A radically different approach is needed, with complementary interventions, including improved water supplies and sanitation, behavior changes, and vector controls, as well as an effective vaccine against schistosomiasis [36].

Observational analyses addressing the effects of helminths remain limited by confounding by poverty and environment. Our strategy aimed to pinpoint helminth effects by randomizing their treatment, but was constrained by difficulties in achieving removal. Trials designed so that helminths are cleared in settings where reinfection can be avoided, and with substantial follow-up, are needed for a full understanding of the risks and benefits of deworming.

## Supplementary Data

Supplementary materials are available at *Clinical Infectious Diseases* online. Consisting of data provided by the authors to benefit the reader, the posted materials are not copyedited and are the sole responsibility of the authors, so questions or comments should be addressed to the corresponding author.

Supplementary Information

## Notes

The LaVIISWA trial team project leaders, physicians, and post-doctoral scientists are Richard Sanya, Margaret Nampijja, Harriet Mpairwe, Geraldine O’Hara, and Barbara Nerima; the statisticians and data managers are Emily Webb, Remy Hoek Spaans, Lawrence Muhangi, Lawrence Lubyayi, Helen Akurut, Fatuma Nalukenge, Beatrice Mirembe, Justin Okello, Sebastian Owilla, Jonathan Levin, and Stephen Nash; the clinical officers are Milly Namutebi and Christopher Zziwa; the nurses are Esther Nakazibwe, Josephine Tumusiime, Caroline Ninsiima, Susan Amongi, Grace Kamukama, Susan Iwala, and Florence Akello; the internal monitor is Mirriam Akello; the field workers are Robert Kizindo, Moses Sewankambo, and Denis Nsubuga; the laboratory staff and collaborators are Stephen Cose, Linda Wammes, Prossy Kabuubi Nakawungu, Emmanuel Niwagaba, Gloria Oduru, Grace Kabami, Elson Abayo, Eric Ssebagala, Fred Muwonge Kakooza, Joyce Kabagenyi, Gyaviira Nkurunungi, Angela Nalwoga, Dennison Kizito, John Vianney Tushabe, Jacent Nassuuna, Jaco Verweij, Serge Versteeg, and Ronald van Ree; the social scientist is Edward Tumwesige; the ultrasonographer is Simon Mpooya; the boatman is David Abiriga; the driver is Richard Walusimbi; human immunodeficiency virus counseling and testing is provided by Victoria Nannozi and Cynthia Kabonesa; the Vector Control Programme staff are James Kaweesa and Edridah Tukahebwa; the administrative management is done by Moses Kizza; and the principal investigator is Alison Elliott.

***Acknowledgments.*** The authors thank the Koome sub-county community members, their local council leaders and beach management committee members, and the village health team members for participating in this study. They thank Koome Health Centre III for their support. They thank the leadership of Mukono District, particularly the district health officer (Elly Tumushabe) and the councillor for Koome sub-county (Asuman Muwumuza), who are members of the Trial Steering Committee, for their support. They also thank the other members of the Trial Steering Committee: Heiner Grosskurth (chair), Edridah Tukahebwa, Narcis Kabatereine, Neil Pearce, Anatoli Kamali, and Monica Kuteesa. They thank the Makerere University Joint AIDS Programme and the Calvary Chapel Island Mission for providing voluntary counselling and human immunodeficiency virus testing for community members, including study participants, in collaboration with their research program. They thank colleagues, especially Richard Hayes, Narcis Kabatereine, and Heiner Grosskurth, for their comments on the manuscript.

***Disclaimer.*** The funders had no role in the study design, data collection and analysis, decision to publish, or preparation of the manuscript.

A. M. E. conceived the study. A. M. E., E. L. W., E. T., R. E. S., M. N., and H. M. participated in designing the trial. R. E. S., A. M. E., M. N., G. O’Hara, C. Z., R. K., J. Kaweesa, E. Nakazibwe, J. T., and F. M. K. led and participated in the surveys. G. Oduru, P. K. N., E. A., and E. Niwagaba ran the field laboratory, while G. N. and J. Kabagenyi participated in establishing and conducting immunological assays and polymerase chain reactions. J. V. trained and assisted in stool polymerase chain reactions. R. vR. contributed to the testing of allergen-specific responses. M. A. monitored the study. L. L. managed the database. E. L. W., S. N., and R. H. S. conducted the statistical analysis. R. E. S., G. N., R. H. S., E. L. W., and A. M. E. drafted the manuscript, and all authors reviewed and contributed to it. All authors read and approved the final version of the manuscript.

***Financial support.*** This work was supported by the Wellcome Trust (grant number 095778). The work was conducted at the Medical Research Council/Uganda Virus Research Institute and London School of Hygiene and Tropical Medicine Uganda Research Unit, which is jointly funded by the UK Medical Research Council and the UK Department for International Development under their Concordat agreement.

***Potential conflicts of interest.*** R. E. S. is supported by a PhD fellowship awarded under the Developing Excellence in Leadership, Training and Science (DELTAS) Africa Initiative (grant number 107743), an independent funding scheme of the African Academy of Sciences and the Alliance for Accelerating Excellence in Science in Africa, and supported by the New Partnership for Africa’s Development Planning and Coordinating Agency with funding from the Wellcome Trust (grant number 107743) and the UK Government. G. N. is supported by a PhD fellowship from the African Partnership for Chronic Disease Research. E. L. W. received salary funding from the Medical Research Council (grant number MR/K012126/1): this award is jointly funded by the UK Medical Research Council and the UK Department for International Development under their Concordat agreement and is also part of the European & Developing Countries Clinical Trials Partnership (EDCTP2) program, supported by the European Union. R. vR. reports personal fees from HAL Allergy BV, Citeq BV, and ThermoFisher Scientific outside the submitted work. All other authors report no potential conflicts. All authors have submitted the ICMJE Form for Disclosure of Potential Conflicts of Interest. Conflicts that the editors consider relevant to the content of the manuscript have been disclosed.
