# Supplementary material for: The Impact of Intensive Versus Standard Anthelminthic Treatment on Allergy-related Outcomes, Helminth Infection Intensity, and Helminth-related Morbidity in Lake Victoria Fishing Communities, Uganda: Results From the LaVIISWA Cluster-randomized Trial
Source: Clin Infect Dis. 2018 Sep 8;68(10):1665–74. doi: 10.1093/cid/ciy761 (PMC6495012; doi:10.1093/cid/ciy761)
Supplement: Supplementary Information [file ciy761_suppl_supplementary_information.docx]

**Supplementary Methods**

***Outcome assessment***

Recent wheeze was assessed by ISAAC (International Study of Asthma and Allergies in Childhood) [1] questionnaire. Visible flexural dermatitis was assessed by using interview questions adopted from the UK diagnostic criteria on atopic eczema and by direct physical examination. Staff were trained in diagnosis of visible flexural dermatitis using the on-line tool by Williams [2]. Pictures of lesions were taken for a second opinion. SPT reactivity to allergens (ALK-Abelló, supplied by Laboratory Specialities (Pty) Ltd, South Africa) was assessed using standard methods. SPT outcomes were examined as any positive response versus none and as positive versus negative for individual allergens. Ultrasonography was performed using a portable machine (*Aloka SSD-900*, 3.5 mH curvilinear probe, Hitachi Aloka Medical, Japan) using the Niamey protocol [3].

Total IgE and asIgE (*D. pteronyssinus*- and *B. germanica*-specific) were measured by ImmunoCAP® (Phadia AB, Sweden) in a random sample of 780 participants (30 per village; selected from those with SPT data and adequate sample volume) and all participants by in-house ELISA [4]. ImmunoCAP outcomes were examined as any positive response versus none (using the standard cutoff of 0.35kUa/L), as positive versus negative for individual allergens, and as continuous variables.

Intestinal helminth infection was investigated by the Kato-Katz method (two slides per sample, read by different technicians) [5] and by stool PCR for *S. mansoni,* *Strongyloides stercoralis* and *Necator americanus* [6]. Urine was assessed for circulating cathodic antigen (CCA, Rapid Medical Diagnostics, South Africa) of *S. mansoni*. *S. haematobium* is not present in the study area [7]. *Mansonella perstans* was determined by the modified Knott’s method [8], malaria by thick film. Voluntary HIV counselling and testing was offered to all participants. Haemoglobin was determined by HemoCue® (HemoCue AB, Angelholm, Sweden).

***Randomisation: further details***

A computer-based system was used to generate all possible allocations for assigning 13 villages to each arm of the trial, satisfying restriction criteria. A list of 1000 of these possible allocations, selected at random from the full list, was provided for the randomisation ceremony. One of these was selected by village leaders using numbered balls drawn from an opaque bag (ten balls, numbered 0 to 9, selected separately for each digit).

***Sample size justification***

For the final survey, we planned to sample 1540 individuals per arm. Based on baseline survey data [9], estimates for primary outcome prevalence in the standard arm were recent wheeze over the age of five years 5%, SPT positivity 20% and detectable asIgE 40%. Assuming a conservative coefficient of variation (k) of 0.3, a sample size of 1540 individuals per arm would give over 80% power to detect (at 5% significance level) a 1.5-fold difference for SPT positivity and detectable asIgE and a 1.8-fold difference for wheeze. From the baseline survey, we calculated that randomly selecting 70 households per village would yield the planned sample size (allowing for absenteeism).

***Approach for adjusted analysis of trial outcomes***

Adjusted analysis was done using a two-stage approach as described by Hayes and Moulton. For binary outcomes, in the first stage logistic regression of individual-level outcomes was done, including covariates to be adjusted for (but not trial arm allocation) and from this risk ratio-residuals (the ratio between observed and predicted prevalence for each cluster) were calculated. In the second stage, these risk ratio-residuals were used as the data points for the cluster-level analysis, using the same methods as described above for the unadjusted analysis. For continuous outcomes, For the two-stage adjusted analysis, linear regression was used in the first stage and mean difference-residuals were data points for the second stage.

**Supplementary Results**

***Comparison of characteristics of survey participants and non-participants***

There were 3566 household members aged one year or above, resident in the 1419 participating households. Of these males were less likely to participate in the questionnaire than females (90% versus 97%), or to give blood (80% versus 87%), stool (75% versus 81%), urine (79% versus 84%) samples or undergo SPT examination (82% versus 89%) (all p<0.001). In both arms low numbers of school-aged children were surveyed because of absence, on the mainland, for schooling. Of those surveyed, working age (15-45 years) and pre-school aged (under 5 years) members were less likely to provide samples or undergo SPT (p<0.001) than other age groups (working age 81%, 74% and 82% for blood, stool and SPT, respectively; pre-school age 74%, 78% and 87%, respectively; other age groups 89%, 86% and 92%, respectively). Younger age groups were under-represented in the subgroup for whom ImmunoCAP assays were done (162 (12%) of <20 year olds versus 618 (28%) of ≥20 year olds, p<0.001), due to lower amounts of available plasma.

**Supplementary Figure. Trial treatment and survey timeline**

**Supplementary Table 1. Impact of intensive versus standard anthelminthic treatment on primary outcomes, restricting to those who had lived in their village throughout (or been born into their village during) the three-year intervention period (“per protocol” analysis)**

|  |  | n/N (%) / geometric mean | |  | Unadjusted | |  | Adjusted for outcome at baseline, age and sex^5^ | |
| --- | --- | --- | --- | --- | --- | --- | --- | --- | --- |
| Outcome | | Standard | Intensive |  | RR/GMR  (95% CI) | p-value |  | RR/GMR  (95% CI) | p-value |
|  | Wheeze (age≥5 years)^1^ | 29/956 (3.0%) | 36/960 (3.8%) |  | 1.23 (0.75, 2.02) | 0.40 |  | 1.14 (0.66, 1.95) | 0.63 |
|  | Wheeze (age<5 years) | 6/234 (2.6%) | 3/210 (1.4%) |  |  |  |  |  |  |
| Atopy (SPT) | |  |  |  |  |  |  |  |  |
|  | SPT positivity to any allergen | 194/1065 (18.2%) | 220/1063 (20.7%) |  | 1.15 (0.84, 1.57) | 0.37 |  | 1.15 (0.86, 1.55) | 0.33 |
|  | SPT positivity to *Dermatophagoides* | 114/1065 (10.7%) | 126/1063 (11.9%) |  | 1.10 (0.76, 1.59) | 0.59 |  | 1.13 (0.80, 1.61) | 0.47 |
|  | SPT positivity to *Blomia tropicalis* | 71/1065 (6.7%) | 97/1062 (9.1%) |  | 1.41 (0.88, 2.24) | 0.14 |  | 1.44 (0.91, 2.26) | 0.11 |
|  | SPT positivity to German cockroach | 106/1064 (10.0%) | 144/1062 (13.6%) |  | 1.41 (0.95, 2.07) | 0.07 |  | 1.38 (0.94, 2.02) | 0.08 |
| Atopy (IgE detected by ImmunoCAP) | |  |  |  |  |  |  |  |  |
|  | *Dermatophagoides* or cockroach positivity (>0.35kUa/L) | 157/280 (56.1%) | 146/259 (56.4%) |  | 1.00 (0.80, 1.24) | 0.97 |  | 0.99 (0.79, 1.24) | 0.95 |
|  | *Dermatophagoides* positivity (asIgE>0.35kUa/L) | 105/280 (37.5%) | 94/259 (36.3%) |  | 0.95 (0.75, 1.20) | 0.64 |  | 0.95 (0.75, 1.20) | 0.63 |
|  | German cockroach positivity (asIgE>0.35kUa/L) | 148/280 (52.9%) | 131/259 (50.6%) |  | 0.95 (0.74, 1.21) | 0.63 |  | 0.95 (0.74, 1.21) | 0.66 |
|  | Concentration of asIgE to *Dermatophagoides* (kUa/L)^2^ | GM: 0.170 | GM: 0.149 |  | 0.84 (0.51, 1.37) | 0.46 |  | 0.84 (0.52, 1.37) | 0.47 |
|  | Concentration of asIgE to German cockroach (kUa/L)^2^ | GM: 0.336 | GM: 0.297 |  | 0.84 (0.53, 1.34) | 0.45 |  | 0.86 (0.54, 1.35) | 0.49 |
| Atopy (IgE detected by in house ELISA) | |  |  |  |  |  |  |  |  |
|  | Concentration of asIgE to *Dermatophagoides*^4^ | GM: 59.9 | GM: 74.1 |  | 1.10 (0.33, 3.67) | 0.87 |  | 1.18 (0.37, 3.76) | 0.77 |
|  | Concentration of asIgE to German cockroach^4^ | GM: 56.9 | GM: 155 |  | 2.05 (0.59, 7.17) | 0.25 |  | 1.63 (0.47, 5.72) | 0.43 |

^1^For this outcome, a natural log transformation was applied to village level proportions to correct skewed distributions; ^2^ log10(+0.001) transformation at individual level; ^3^ log10(+1) transformation at individual level; ^4^log10(+1) transformation at individual level; ^5^Atopy outcomes assessed by IgE were adjusted for age and sex only; RR: risk ratio; GM: geometric mean; GMR: geometric mean ratio; CI: confidence interval

**Supplementary Table 2. Impact of intensive versus standard anthelminthic treatment on primary outcomes, stratified by age group (<4 years, ≥4 years)**

|  |  | <4 years | |  | ≥4 years | |  |  |
| --- | --- | --- | --- | --- | --- | --- | --- | --- |
| Outcome | | RR/GMR (95% CI) | p-value |  | RR/GMR (95% CI) | p-value |  | Interaction  p-value |
| Atopy (SPT) | | (n=382) |  |  | (n=2655) |  |  |  |
|  | SPT positivity to any allergen | 1.63 (0.68, 3.91) | 0.23 |  | 1.07 (0.81, 1.41) | 0.63 |  | 0.57 |
|  | SPT positivity to *Dermatophagoides* | 1.49 (0.41, 5.34) | 0.51 |  | 0.97 (0.71, 1.33) | 0.84 |  | 0.48 |
|  | SPT positivity to *Blomia tropicalis* | 1.44 (0.48, 4.27) | 0.50 |  | 1.26 (0.82, 1.94) | 0.28 |  | 0.83 |
|  | SPT positivity to German cockroach | 2.12 (0.37, 12.32) | 0.35 |  | 1.22 (0.86, 1.73) | 0.24 |  | 0.79 |
| Atopy (IgE detected by ImmunoCAP) | | (n=44) |  |  | (n=736) |  |  |  |
|  | *Dermatophagoides* or cockroach positivity (>0.35kUa/L) | 1.38 (0.26, 7.34) | 0.72 |  | 0.96 (0.84, 1.11) | 0.60 |  | 0.69 |
|  | *Dermatophagoides* positivity (asIgE>0.35kUa/L) | 0.55 (0.05, 6.73) | 0.61 |  | 0.95 (0.76, 1.19) | 0.65 |  | 0.88 |
|  | German cockroach positivity (asIgE>0.35kUa/L) | 2.41 (0.37, 15.71) | 0.41 |  | 0.93 (0.80, 1.10) | 0.39 |  | 0.39 |
|  | Concentration of asIgE to *Dermatophagoides* (kUa/L) | 0.43 (0.10, 1.80) | 0.24 |  | 0.77 (0.50, 1.17) | 0.21 |  | 0.43 |
|  | Concentration of asIgE to German cockroach (kUa/L) | 0.95 (0.27, 3.30) | 0.93 |  | 0.80 (0.55, 1.16) | 0.22 |  | 0.91 |
| Atopy (IgE detected by in house ELISA) | | (n=309) |  |  | (n=2646) |  |  |  |
|  | Concentration of asIgE to *Dermatophagoides* | 0.94 (0.18, 4.98) | 0.94 |  | 1.17 (0.38, 3.58) | 0.78 |  | 0.70 |
|  | Concentration of asIgE to German cockroach | 1.77 (0.36, 8.69) | 0.46 |  | 2.03 (0.63, 6.53) | 0.23 |  | 0.69 |

**Supplementary Table 3. Impact of intensive versus standard anthelminthic treatment on schistosomiasis-related liver and spleen morbidity assessed by ultrasonography, in children<18 years**

|  | n (%) | |  | Unadjusted | |  | Adjusted for age and sex | |
| --- | --- | --- | --- | --- | --- | --- | --- | --- |
| Outcome | Standard (n=414) | Intensive (n=488) |  | OR (95% CI) | p-value |  | OR (95% CI) | p-value |
| **Liver size left lobe^1^** |  |  |  |  |  |  |  |  |
| Normal | 171 (41.3%) | 212 (43.4%) |  |  |  |  |  |  |
| Enlarged | 212 (51.2%) | 229 (46.9%) |  |  |  |  |  |  |
| Much enlarged | 31 (7.5%) | 47 (9.6%) |  | 0.97 (0.58, 1.61) | 0.90 |  | 1.05 (0.63, 1.72) | 0.86 |
| **Spleen size^1^** |  |  |  |  |  |  |  |  |
| Normal | 248 (59.9%) | 302 (61.9%) |  |  |  |  |  |  |
| Moderate splenomegaly | 135 (32.6%) | 142 (29.1%) |  |  |  |  |  |  |
| Marked splenomegaly | 31 (7.5%) | 44 (9.0%) |  | 0.87 (0.40, 1.89) | 0.71 |  | 0.86 (0.40, 1.86) | 0.69 |
| **Portal vein score^2^** |  |  |  |  |  |  |  |  |
| Normal | 319 (77.1%) | 383 (78.5%) |  |  |  |  |  |  |
| Dilation | 94 (22.7%) | 103 (21.1%) |  |  |  |  |  |  |
| Marked dilation | 1 (0.2%) | 2 (0.4%) |  | 0.70 (0.40, 1.24) | 0.23 |  | 0.84 (0.50, 1.42) | 0.51 |
| **Degree of hepatic fibrosis^3^** |  |  |  |  |  |  |  |  |
| Normal | 386 (93.7%) | 445 (91.4%) |  |  |  |  |  |  |
| Peri-portal fibrosis | 26 (6.3%) | 42 (8.6%) |  | 1.52 (0.75, 3.07) | 0.26 |  | 1.45 (0.72, 2.94) | 0.31 |

Measurements from ultrasonography were classified according to the Niamey protocol, taking height into account; ^1^For liver size left lobe and spleen size, ORs were calculated using a proportional odds model; ^2^Portal vein score was analysed as a binary variable, combining dilated and marked dilated categories; ^3^Degree of hepatic fibrosis was classified as normal (Niamey protocol image pattern A) or peri-portal fibrosis (Niamey protocol image patterns B-F) and analysed as a binary variable, degree of hepatic fibrosis missing for 2 children in standard arm, 1 child in intensive arm

**Supplementary Table 4. Impact of intensive versus standard anthelminthic treatment on helminths, clinical outcomes, hepatosplenomegaly by palpation, and anthropometry, restricting to those who had lived in their village throughout (or been born into their village during) the three-year intervention period (“per protocol” analysis)**

|  | | n/N (%) / arithmetic mean | |  | Unadjusted | |  | Adjusted for baseline value, age and sex | |
| --- | --- | --- | --- | --- | --- | --- | --- | --- | --- |
| Outcome | | Standard | Intensive |  | RR/mean difference (95% CI) | p-value |  | RR/mean difference (95% CI) | p-value |
| Helminth infections | |  |  |  |  |  |  |  |  |
|  | *Schistosoma mansoni*, stool Kato Katz | 373/965 (38.7%) | 225/989 (22.8%) |  | 0.62 (0.41, 0.94) | 0.02 |  | 0.69 (0.53, 0.90) | 0.005 |
|  | *Schistosoma mansoni, stool PCR* | 576/964 (59.8%) | 367/987 (37.2%) |  | 0.65 (0.48, 0.88) | 0.006 |  | 0.73 (0.62, 0.87) | 0.001 |
|  | *Schistosoma mansoni*, urine CCA | 872/1015 (85.9%) | 838/1002 (83.6%) |  | 0.98 (0.90, 1.07) | 0.61 |  | 0.99 (0.92, 1.07) | 0.81 |
|  | Hookworm, stool PCR^1^ | 84/964 (8.7%) | 62/987 (6.3%) |  | 0.56 (0.27, 1.19) | 0.12 |  | 0.58 (0.29, 1.13) | 0.10 |
|  | *Strongyloides stercoralis*, stool PCR | 84/964 (8.7%) | 51/987 (5.2%) |  | 0.63 (0.40, 0.99) | 0.04 |  | 0.68 (0.44, 1.04) | 0.07 |
|  | *Trichuris trichiura*, stool Kato Katz^1^ | 100/965 (10.4%) | 79/989 (8.0%) |  | 0.84 (0.38, 1.84) | 0.64 |  | 0.77 (0.48, 1.23) | 0.26 |
|  | *Ascaris lumbricoides*, stool Kato Katz | 8/965 (0.8%) | 3/989 (0.3%) |  |  |  |  |  |  |
| Clinical outcomes | |  |  |  |  |  |  |  |  |
|  | Visible flexural dermatitis | 1/1099 (0.1%) | 1/1085 (0.1%) |  |  |  |  |  |  |
|  | Haemoglobin | 14.0 | 13.9 |  | -0.12 (-0.47, 0.22) | 0.48 |  | -0.01 (-0.25, 0.24) | 0.96 |
| Anthropometry | |  |  |  |  |  |  |  |  |
|  | Height-for-age z-score, age 1-19 years | -0.39 | -0.42 |  | 0.05 (-0.21, 0.30) | 0.71 |  | 0.09 (-0.16, 0.34) | 0.45 |
|  | Weight-for-age z-score, age 1-10 years | -0.09 | -0.13 |  | -0.08 (-0.31, 0.16) | 0.51 |  | -0.05 (-0.23, 0.12) | 0.52 |
|  | Weight-for-height z-score, age 1-5 years | 0.12 | 0.16 |  | -0.08 (-0.47, 0.30) | 0.65 |  | -0.06 (-0.44, 0.31) | 0.73 |
| Hepatosplenomegaly, palpation | |  |  |  |  |  |  |  |  |
|  | Hepatomegaly, palpation | 84/1088 (7.7%) | 78/1080 (7.2%) |  | 0.93 (0.65, 1.33) | 0.69 |  | 0.93 (0.64, 1.35) | 0.68 |
|  | Splenomegaly, palpation | 59/1090 (5.4%) | 40/1081 (3.7%) |  | 0.72 (0.40, 1.31) | 0.22 |  | 0.72 (0.40, 1.30) | 0.22 |
|  | Hepatosplenomegaly, palpation^1^ | 14/1089 (1.3%) | 8/1082 (0.7%) |  |  |  |  |  |  |
| Reported clinical outcomes (exploratory) | |  |  |  |  |  |  |  |  |
|  | Urticaria, last 12 months | 108/1190 (9.1%) | 127/1170 (10.9%) |  | 1.20 (0.91, 1.58) | 0.19 |  | 1.20 (0.94, 1.53) | 0.13 |
|  | Rhinitis, last 12 months | 58/1190 (4.9%) | 47/1170 (4.0%) |  | 0.89 (0.60, 1.32) | 0.56 |  | 0.87 (0.58, 1.31) | 0.50 |

^1^ For this outcome, a natural log transformation was applied to village level proportions to correct skewed distributions; RR: Risk Ratio; CI: confidence interval; CCA: circulating cathodic antigen; PCR: polymerase chain reaction

**Supplementary Table 5: CONSORT 2010 checklist of information to include when reporting a cluster randomised trial**

| Section/Topic | Item No | Standard Checklist item | Extension for cluster designs | Page No * |
| --- | --- | --- | --- | --- |
| Title and abstract | | | |  |
|  | 1a | Identification as a randomised trial in the title | Identification as a cluster randomised trial in the title | Page 1 |
|  | 1b | Structured summary of trial design, methods, results, and conclusions (for specific guidance see CONSORT for abstracts) | See table 2 | Pages 5-6 |
| Introduction | | | |  |
| Background and objectives | 2a | Scientific background and explanation of rationale | Rationale for using a cluster design | Pages 7-8 |
|  | 2b | Specific objectives or hypotheses | Whether objectives pertain to the cluster level, the individual participant level or both | Pages 7-8 |
| Methods | | | |  |
| Trial design | 3a | Description of trial design (such as parallel, factorial) including allocation ratio | Definition of cluster and description of how the design features apply to the clusters | Page 8 |
|  | 3b | Important changes to methods after trial commencement (such as eligibility criteria), with reasons |  | NA |
| Participants | 4a | Eligibility criteria for participants | Eligibility criteria for clusters | Pages 8-9 |
|  | 4b | Settings and locations where the data were collected |  | Page 8 |
| Interventions | 5 | The interventions for each group with sufficient details to allow replication, including how and when they were actually administered | Whether interventions pertain to the cluster level, the individual participant level or both | Page 8 |
| Outcomes | 6a | Completely defined pre-specified primary and secondary outcome measures, including how and when they were assessed | Whether outcome measures pertain to the cluster level, the individual participant level or both | Pages 9-10, Supplementary Methods |
|  | 6b | Any changes to trial outcomes after the trial commenced, with reasons |  | Page 10 |
| Sample size | 7a | How sample size was determined | Method of calculation, number of clusters(s) (and whether equal or unequal cluster sizes are assumed), cluster size, a coefficient of intracluster correlation (ICC or *k*), and an indication of its uncertainty | Page 10, Supplementary Methods |
|  | 7b | When applicable, explanation of any interim analyses and stopping guidelines |  | N/A |
| Randomisation: | | | |  |
| Sequence generation | 8a | Method used to generate the random allocation sequence |  | Page 10, Supplementary Methods |
|  | 8b | Type of randomisation; details of any restriction (such as blocking and block size) | Details of stratification or matching if used | Page 10, Supplementary Methods |
| Allocation concealment mechanism | 9 | Mechanism used to implement the random allocation sequence (such as sequentially numbered containers), describing any steps taken to conceal the sequence until interventions were assigned | Specification that allocation was based on clusters rather than individuals and whether allocation concealment (if any) was at the cluster level, the individual participant level or both | Page 10, Supplementary Methods |
| Implementation | 10 | Who generated the random allocation sequence, who enrolled participants, and who assigned participants to interventions | Replace by 10a, 10b and 10c |  |
|  | 10a |  | Who generated the random allocation sequence, who enrolled clusters, and who assigned clusters to interventions | Page 10, Supplementary Methods |
|  | 10b |  | Mechanism by which individual participants were included in clusters for the purposes of the trial (such as complete enumeration, random sampling) | Pages 9-10 |
|  | 10c |  | From whom consent was sought (representatives of the cluster, or individual cluster members, or both), and whether consent was sought before or after randomisation | Page 9 |
|  |  |  |  |  |
| Blinding | 11a | If done, who was blinded after assignment to interventions (for example, participants, care providers, those assessing outcomes) and how |  | N/A |
|  | 11b | If relevant, description of the similarity of interventions |  | N/A |
| Statistical methods | 12a | Statistical methods used to compare groups for primary and secondary outcomes | How clustering was taken into account | Pages 10-11, Supplementary Methods |
|  | 12b | Methods for additional analyses, such as subgroup analyses and adjusted analyses |  | Page 11, Supplementary Methods |
| Results | | | |  |
| Participant flow (a diagram is strongly recommended) | 13a | For each group, the numbers of participants who were randomly assigned, received intended treatment, and were analysed for the primary outcome | For each group, the numbers of clusters that were randomly assigned, received intended treatment, and were analysed for the primary outcome | Pages 11-12, Figure 2 |
|  | 13b | For each group, losses and exclusions after randomisation, together with reasons | For each group, losses and exclusions for both clusters and individual cluster members | N/A |
| Recruitment | 14a | Dates defining the periods of recruitment and follow-up |  | Pages 11-12 |
|  | 14b | Why the trial ended or was stopped |  | N/A |
| Baseline data | 15 | A table showing baseline demographic and clinical characteristics for each group | Baseline characteristics for the individual and cluster levels as applicable for each group | Table 1 |
| Numbers analysed | 16 | For each group, number of participants (denominator) included in each analysis and whether the analysis was by original assigned groups | For each group, number of clusters included in each analysis | Figure 2, Tables 2 and 3, Page 12 |
| Outcomes and estimation | 17a | For each primary and secondary outcome, results for each group, and the estimated effect size and its precision (such as 95% confidence interval) | Results at the individual or cluster level as applicable and a coefficient of intracluster correlation (ICC or k) for each primary outcome | Pages 12-13, Tables 2 and 3, Supplementary Table 3 |
|  | 17b | For binary outcomes, presentation of both absolute and relative effect sizes is recommended |  |  |
| Ancillary analyses | 18 | Results of any other analyses performed, including subgroup analyses and adjusted analyses, distinguishing pre-specified from exploratory |  | Pages 12-13, Supplementary Tables 1, 2 and 4 |
| Harms | 19 | All important harms or unintended effects in each group (for specific guidance see CONSORT for harms) |  | Pages 13-14 |
| Discussion | | | |  |
| Limitations | 20 | Trial limitations, addressing sources of potential bias, imprecision, and, if relevant, multiplicity of analyses |  | Pages 14-16 |
| Generalisability | 21 | Generalisability (external validity, applicability) of the trial findings | Generalisability to clusters and/or individual participants (as relevant) | Pages 15-16 |
| Interpretation | 22 | Interpretation consistent with results, balancing benefits and harms, and considering other relevant evidence |  | Pages 14-16 |
| Other information | | |  |  |
| Registration | 23 | Registration number and name of trial registry |  | Page 5 |
| Protocol | 24 | Where the full trial protocol can be accessed, if available |  | Page 8 |
| Funding | 25 | Sources of funding and other support (such as supply of drugs), role of funders |  | Page 17 |

** Note: page numbers optional depending on journal requirements*

1. Amoah AS, Obeng BB, Larbi IA, Versteeg SA, Aryeetey Y, Akkerdaas JH, et al. Peanut-specific IgE antibodies in asymptomatic Ghanaian children possibly caused by carbohydrate determinant cross-reactivity. The Journal of allergy and clinical immunology. 2013;132(3):639-47. Epub 2013/06/15. doi: 10.1016/j.jaci.2013.04.023. PubMed PMID: 23763976; PubMed Central PMCID: PMCPMC3765958.

2. Williams HC. So how do I define Atopic Eczema? A practical manual for researchers wishing to define atopic eczema [15th September 2010]. Available from: http://www.nottingham.ac.uk/dermatology/eczema/index.html.

3. WHO. Ultrasound in schistosomiasis: a practical guide to the standardized use of ultrasonography for the assessment of schistosomiasis-related morbidity2000 28th May 2017. Available from: <http://apps.who.int/iris/bitstream/10665/66535/1/TDR_STR_SCH_00.1.pdf>.

4. Mpairwe H, Webb EL, Muhangi L, Ndibazza J, Akishule D, Nampijja M, et al. Anthelminthic treatment during pregnancy is associated with increased risk of infantile eczema: randomised-controlled trial results. Pediatric allergy and immunology : official publication of the European Society of Pediatric Allergy and Immunology. 2011;22(3):305-12. Epub 2011/01/25. doi: 10.1111/j.1399-3038.2010.01122.x [doi]. PubMed PMID: 21255083.

5. Katz N, Chaves A, Pellegrino J. A simple device for quantitative stool thick-smear technique in Schistosomiasis mansoni. Rev Inst Med Trop Sao Paulo. 1972;14(6):397-400. Epub 1972/11/01. PubMed PMID: 4675644.

6. Verweij JJ, Canales M, Polman K, Ziem J, Brienen EA, Polderman AM, et al. Molecular diagnosis of Strongyloides stercoralis in faecal samples using real-time PCR. Transactions of the Royal Society of Tropical Medicine and Hygiene. 2009;103(4):342-6. Epub 2009/02/07. doi: 10.1016/j.trstmh.2008.12.001. PubMed PMID: 19195671.

7. Emmanuel IO, Ekkehard D. Epidemiology, of bilharzias (schistosomiasis) in Uganda from 1902 until 2005. African health sciences. 2008;8(4):239-43. Epub 2008/12/01. PubMed PMID: 20589131; PubMed Central PMCID: PMCPMC2887015.

8. Melrose WD, Turner PF, Pisters P, Turner B. An improved Knott's concentration test for the detection of microfilariae. Transactions of the Royal Society of Tropical Medicine and Hygiene. 2000;94(2):176. Epub 2000/07/18. PubMed PMID: 10897361.

9. Nampijja M, Webb EL, Kaweesa J, Kizindo R, Namutebi M, Nakazibwe E, et al. The Lake Victoria Island Intervention Study on Worms and Allergy-related diseases (LaVIISWA): study protocol for a randomised controlled trial. Trials. 2015;16:187. Epub 2015/04/24. doi: 10.1186/s13063-015-0702-5. PubMed PMID: 25902705; PubMed Central PMCID: PMC4413531.
